# The complexity of gene expression dynamics revealed by permutation entropy

**DOI:** 10.1186/1471-2105-11-607

**Published:** 2010-12-22

**Authors:** Xiaoliang Sun, Yong Zou, Victoria Nikiforova, Jürgen Kurths, Dirk Walther

**Affiliations:** 1 Max Planck Institute for Molecular Plant Physiology, Am Mühlenberg 1, 14476 Potsdam-Golm, Germany; 2 Potsdam Institute for Climate Impact Research, P.O. Box 60120314412 Potsdam, Germany; 3 Department of Physics, Humboldt University Berlin, Newtonstraße 1512489 Berlin, Germany; 4 Institute of Complex Systems and Mathematical Biology, University of Aberdeen, Aberdeen, UK; 5 Molecular Systems Biology, University of Vienna, Althanstr. 14 1090 Vienna, Austria

## Abstract

**Background:**

High complexity is considered a hallmark of living systems. Here we investigate the complexity of temporal gene expression patterns using the concept of Permutation Entropy (PE) first introduced in dynamical systems theory. The analysis of gene expression data has so far focused primarily on the identification of differentially expressed genes, or on the elucidation of pathway and regulatory relationships. We aim to study gene expression time series data from the viewpoint of complexity.

**Results:**

Applying the PE complexity metric to abiotic stress response time series data in Arabidopsis thaliana, genes involved in stress response and signaling were found to be associated with the highest complexity not only under stress, but surprisingly, also under reference, non-stress conditions. Genes with house-keeping functions exhibited lower PE complexity. Compared to reference conditions, the PE of temporal gene expression patterns generally increased upon stress exposure. High-complexity genes were found to have longer upstream intergenic regions and more cis-regulatory motifs in their promoter regions indicative of a more complex regulatory apparatus needed to orchestrate their expression, and to be associated with higher correlation network connectivity degree. Arabidopsis genes also present in other plant species were observed to exhibit decreased PE complexity compared to Arabidopsis specific genes.

**Conclusions:**

We show that Permutation Entropy is a simple yet robust and powerful approach to identify temporal gene expression profiles of varying complexity that is equally applicable to other types of molecular profile data.

## Background

High complexity is considered a defining feature setting living systems apart from non-living matter. Thus, investigating the emergence, maintenance, and functional significance of complexity has been a central research theme in all of biological sciences throughout history including the analysis of the complexity of cellular systems at the molecular level [1] and culminating in the emergence of Systems Biology that aims to develop an holistic understanding of the complex behavior of molecular and cellular systems [2]. In the context of molecular interaction networks, for example, it was observed that eukaryotic evolution was accompanied by changes of the complexity and a fast - on an evolutionary time scale - rewiring of interactions between proteins [3]. However, in the temporal domain, the complexity of molecular processes has not been adequately investigated yet. Dynamic phenomena such as the temporal gene expression response to external perturbations as measured in time course genome-scale microarray measurements, while constituting a major research topic, have been analyzed primarily to unravel structural relationships between different groups of genes with the aim to identify important gene sets - for example, for diagnostic purposes - via clustering [4-8] or principal component analysis (PCA) [6,9], or to deduce regulatory transcriptional networks and modules [10-13], infer relationships between metabolic genes [14,15] as well as to provide a basis for network modeling [16,17]. Along with increasing numbers of experiments involving expression time series, approaches to identify dominating temporal patterns have been developed. Introduced approaches ranged from applying unbiased Singular Value Decomposition (SVD, [18]), utilizing the notion of patterns [19] and extracting gene sets that are consistent with simple up/down/unchanged patterns and successions thereof as a means to guided profile clustering [7], and to converting continuous level values into discrete ranks to determine the degree of randomness with regard to rank permutations [20].

The paucity of systematic studies of temporal gene expression complexity may in part be explained by the lack of a suitable metric that is applicable to the typically very short gene expression time series with only few time points per gene available.

The investigation of complexity has attracted considerable interest in the physical sciences and various other fields. A quantitative understanding of complexity emerging in dynamical systems is often obtained by the notion of entropy, Lyapunov exponents, or fractal dimensions [21]. The former two quantities measure the predictability of a system by showing how sensitively it depends on the initial conditions, while fractal dimension characterizes the complex geometric property in phase space.

It is rather challenging to apply the physical concepts of complexity (e.g., entropy) to address biological complexity, especially in the context of temporal gene expression data. Specifically, the encountered obstacles include: (i) Very short time series. For example, entropy as a complexity measure is formally defined only in the asymptotic limit; i.e., very long time series data at arbitrary accuracy are needed. Finite time records require a suitable modification, e.g. ε-entropy [22]. Still, even this modified entropy definition is not properly applicable to typical biological datasets, especially microarray measurements. Most microarray experiments include a very limited number of temporal measurements, typically no more than 10 or so, oftentimes simply because of the cost considerations. (ii) Non-stationarity. Most microarray measurements are intentionally non-stationary as they are being conducted specifically to measure the system's response to an external perturbation. Note that here only a weak non-stationary criterion is considered, where the mean and the variance might vary over time. (iii) Non-equidistant time scale. Microarray sampling schedules are intentionally designed with denser coverage at early time intervals of the process as the initial phases are, first, most interesting, and secondly, as the new steady state is reached, the rate of molecular adaptation can be expected to slow down.

In an attempt to capture complexity of temporal profiles understood as algorithmic compressibility and to identify profiles that are highly non-random, Ahnert and co-workers introduced a rank permutation-based approach [20]. In this approach, a particular series of level data is converted to ranks and by virtue of various mapping functions that associate patterns with a single number, its likelihood of resulting from a random as opposed to a biological process can be assessed in an unbiased fashion. This method was shown to correctly identify cell cycle genes in yeast without the need to introduce any pre-conceptions on the data.

Here, we propose to use Permutation Entropy (PE) to analyze temporal profile data. PE was initially introduced as a measure to assess the complexity for time series based only on the ranks of the data, instead of a particular distance metric [23]. PE decomposes data series (e.g. expression time series data) into elementary motifs (ordinal or order motifs capturing permutations of ranks) and associates high complexity with high and low complexity with low numbers of different motifs observed in a given temporal profile. So far, it has been applied mainly to detect dynamical transitions in both modeled and experimental data; i.e., electroencephalography (EEG) and magneto-cardiography records [24]. Compared to the approach reported in [20], it has the advantage of only using a single pattern-to-value mapping function. Furthermore, we introduced the 'unchanged' pattern; i.e., differentiate between significant expression changes and those that are purely attributable to noise.

We applied the concept of PE to gene expression time series data obtained in *Arabidopsis thaliana*, an important model plant [25], exposed to different abiotic stress conditions to investigate specifically, how the complexity of temporal gene expression patterns changes upon stress exposure and whether different functional groups of genes as well as different experimental conditions are characterized by different PE properties. Secondly, we explore whether and to what degree gene-specific expression complexity is encoded in the Arabidopsis genome by examining associated gene promoter regions. Whether complexity is increasing during the course of evolution is an intriguing and much debated question [26]. Here, we compare the obtained PE complexity measure for well-conserved (across different plant species) - and thus supposedly ancient - genes to those associated with Arabidopsis-specific, presumably young genes. Finally, we examine the introduced PE complexity measure in relation to gene expression correlation networks. Correlation networks allow studying gene expression data sets with regard to the structure of the underlying pairwise relationships with particular focus on genes involved in many interactions, so-called hub genes with a high degree of network connectivity [27,28]. We find that high-degree genes are associated with increased PE indicative of the global stress induced restructuring of gene expression that has been observed similarly in yeast [29].

We demonstrate that PE is a simple, yet powerful novel concept to study the dynamics of temporal gene expression profiles and equally applicable to other types of molecular profile data.

## Results

We applied the concept of Permutation Entropy (PE) as a metric to assess the complexity of temporal gene expression profiles obtained from abiotic stress time series microarray experiments performed in *Arabidopsis thaliana *[30]. The dataset comprised nine abiotic stress (including heat, cold, genotoxic, UV-B-light, osmotic, salt, wounding, drought, and oxidative stress conditions) and one common control condition. For every stress and the control condition, 7 time points were consistently available across all conditions corresponding to 0 h, 0.5 h, 1 h, 3 h, 6 h, 12 h, and 24 h after stress exposure.

### Elementary three-point pattern distribution

**Figure 1:**
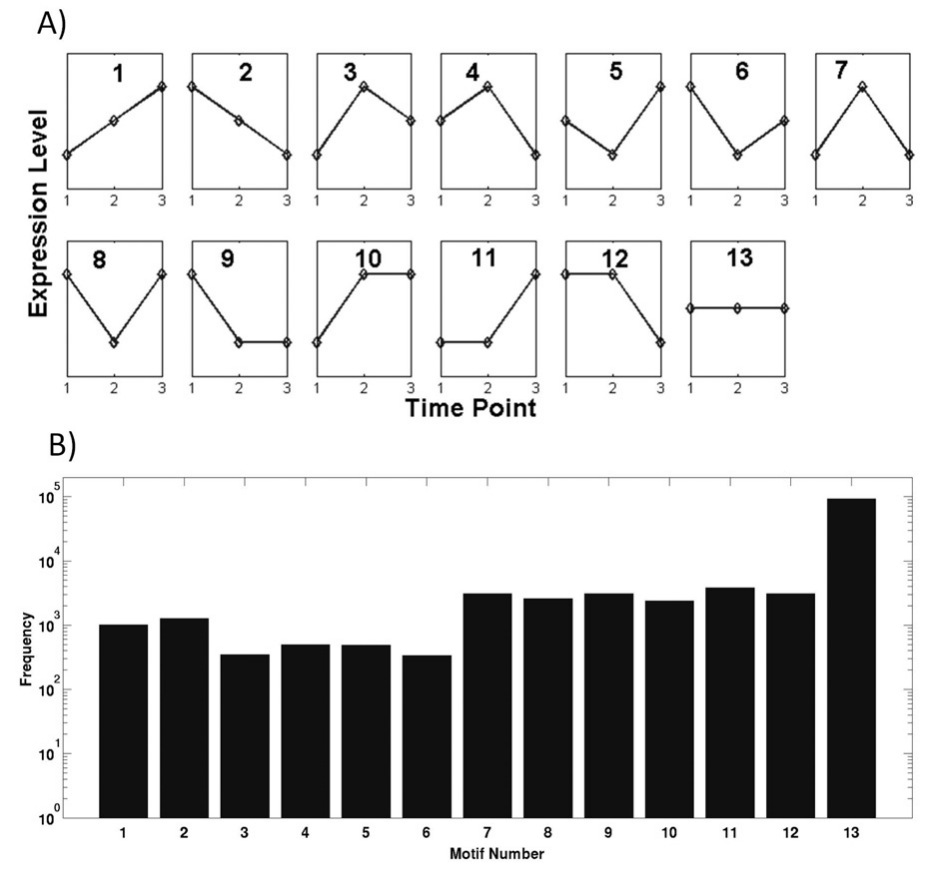
**A) The 13 different possible ordinal patterns (or order patterns/motifs) of consecutive three time-point data**. In each sub-graph, the *x*-axis is the time point and *y*-axis the expression level (in arbitrary units). Note that sub-graphs 9-13 include the no-change pattern introduced here to account for noise-only changes (see Methods). B) Frequency distribution of every order motif across all time-points in the control dataset (21,797 genes times 5 patterns per 7-point time series = 108,985 total motifs) with motif numberings according to A.

The approach to measure complexity of temporal gene expression profiles by means of Permutation Entropy (PE) relies on converting continues expression level data into a succession of discrete order patterns of - in our case - three consecutive time points. Allowing for the no-change pattern (see Methods), 13 different three-point patterns are possible (Figure 1A). The most frequent pattern observed under control conditions and across all gene transcripts is pattern 13, the no-change pattern (Figure 1B), followed by partially unchanged patterns (patterns 7-12) and monotonic profile patterns (pattern 1 and 2). Least frequent are patterns corresponding to three distinct expression values (patterns 3-6). Notwithstanding the fact that the no-change pattern assignment depends on the set expression difference threshold between consecutive time points (*d_e_*, see Methods), a clear tendency to steady or gradually rather than abruptly changing elementary expression patterns is evident from the data.

### The Permutation Entropy (PE) distribution of the Arabidopsis transcriptome under control and stress conditions

**Figure 2:**
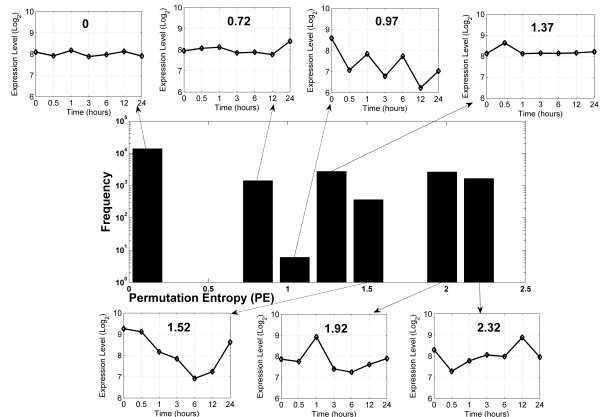
**Distribution of PE values and associated selected expression profile illustrations in the control dataset**. In each sub-graph associated with every discrete PE value, an example gene expression profile is plotted to illustrate the relationship between PE and expression profile.

Applying our modified PE metric to all AtGenExpress abiotic stress time series datasets, the entropy values for all considered 21,797 (22k for short) genes under all 10 different conditions (nine stress and one control condition) were calculated. As an illustration, we selected the control dataset to show the general distribution of PE values across all gene transcripts (Figure 2). As only seven discrete PE values are possible given our definitions (see Methods) represented by the seven frequency bars in Figure 2, the distribution is not normal, and hence, all significance tests were performed applying the Mann-Whitney-U test (MWU-test or, as it is also known, the Wilcoxon rank sum test). The smallest PE value (PE = 0) corresponds to genes with only small fluctuation across all the time points such that gene expression levels do not change above the d_e _threshold (no-change pattern) as illustrated for an exemplary profile surrounding the histogram or to strictly monotonic up or downward changes across all time points as such a profile also yields PE = 0. As evident from the example profiles, with increasing PE values, the associated temporal patterns become more complex; i.e., more and different patterns occur with the maximal possible PE value of 2.32. Zero is the most frequent PE value obtained for approximately half of all gene transcripts; i.e., most genes have a low expression complexity under control conditions. The maximal PE value was obtained for only about 10 percent of all transcripts. The least frequent profiles are the strictly periodic signals (third bar from the left).

Relative to control conditions, the median PE of all 22k gene transcripts monitored by the microarray increases significantly upon exposure to stress conditions (Table 1). The most dramatic increase of PE was observed under heat and UV-B light stress conditions, and smallest changes, but nonetheless leading to significantly increased PE, under cold and oxidative stress situations. Thus, when challenged by unfavorable environmental conditions, the complexity of gene expression programs in Arabidopsis increases, whereas the control condition appears to correspond to the ground state of minimal PE complexity.

**Table 1:** Significance of PE change upon stress exposure.

	Cold	Genotoxic	Osmotic	Salt	UV-B	Wounding	Drought	Heat	Oxidative
**PE_stress_-PE_control_**	0.11	0.12	0.26	0.15	0.46	0.20	0.14	0.48	0.11

**p-value**	3.4e-76	2.6e-61	5.1e-228	3.8e-79	0	3.2e-121	1.5e-68	0	2.2e-48

Differences between the median PE-value of all transcripts under stress compared to median PE under control conditions. *p*-values correspond to the Wilcoxon rank sum test.

### Functional association analysis of high and low PE genes

We examined what biological processes and molecular functions are associated with genes of high or low PE, respectively. Moreover, we asked whether the association of particular processes and functions to high or low PE changes under different stress conditions or whether there are particular functional gene classes universally associated with either high or low PE. All gene transcripts were classified into 14 biological processes and 15 molecular function groups according to GO-slim terms (see Methods) followed by computing the mean PE value per condition for each GO gene set and applying biclustering (clustering conditions and GO terms simultaneously).

**Figure 3:**
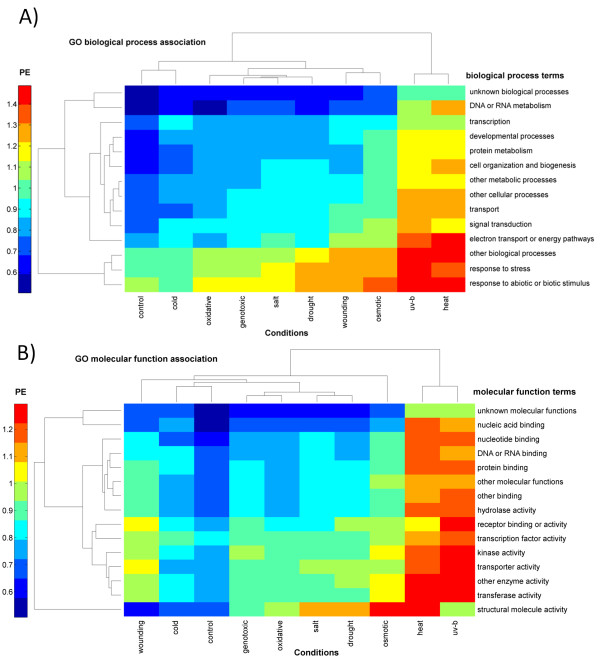
**Biclustering (average linkage) of the mean *PE *computed for gene sets grouped by their GO-Slim biological process (A) or function (B) annotation across all nine abiotic stress conditions and the control condition**.

Across all conditions (including the control condition), genes involved in the general and abiotic stress response (Figure 3A) and regulatory processes (transcription factor activity, kinase activity (Figure 3B)) are universally and significantly (Table 2) associated with high PE (Figure 3A) indicating their sensitivity towards stress conditions, while genes of yet unidentified functions and process involvement and house-keeping functions such as DNA and RNA metabolism (Figure 3A) as well as general transcription processes and functions (DNA and RNA binding, Figure 3B) were found to represent a low PE cluster. Thus, their gene expression is less complex even when exposed to stress conditions. Surprisingly, even under control conditions, response to stress and transcription factor activity are significantly enriched in the high PE gene set suggesting that genes involved in these functions exhibit greater temporal gene expression complexity even under normal reference conditions (Table 2). Nonetheless, the reference, control condition was associated with relatively low PE values across most GO terms, while heat and UV-B light stress clearly stand out as being associated with high PE across all GO categories (Figures 3A and 3B). Interestingly, cold and heat stress exposure appear to fall on opposite ends of the PE spectrum with cold stress being similar to control conditions. Thus, temperature appears to either "freeze" = cold or "stir" up = heat the system reflected by PE. The conditions "salt" and "drought" stress - both water stress related - were found to cluster together based on mean PE values for the different GO terms as well as "oxidative" and "genotoxic" stress, albeit less tightly.

**Table 2:** GO categories associated with high PE.

Conditions	Biological Process	FDR p-value	Molecular Function	FDR p-value
**Control**	response to abiotic or biotic stimulus	2.8e-19	transcription factor activity	3.4e-04
	
	response to stress	1.6e-13	other enzyme activity	2.1e-03
	
	other biological processes	7.7e-10	other molecular functions	4.5e-02

**Cold**	response to stress	4.9e-08	transcription factor activity	1.7e-07
	
	other biological processes	1.3e-07	receptor binding or activity	3.8e-03
	
	response to abiotic or biotic stimulus	8.1e-06	kinase activity	2.9e-02
	
	transcription	3.3e-04		
	
	signal transduction	3.2e-03		

**Genotoxic**	response to abiotic or biotic stimulus	2.9e-20	kinase activity	7.6e-06
	
	response to stress	3.8e-16	transcription factor activity	8.9e-04
	
	other biological processes	1.8e-11	transferase activity	1.7e-03
	
	signal transduction	7.3e-03	transporter activity	1.4e-02

**Osmotic**	response to stress	1.4e-16	transferase activity	7.0e-04
	
	response to abiotic or biotic stimulus	1.4e-14	kinase activity	1.4e-03
	
	other biological processes	1.3e-08	transcription factor activity	1.3e-02
	
	signal transduction	1.3e-02	other molecular functions	4.3e-02

**Salt**	response to abiotic or biotic stimulus	3.0e-12	structural molecule activity	1.1e-13
	
	response to stress	6.0e-07	transferase activity	2.3e-03
	
	other biological processes	3.3e-03	other enzyme activity	2.4e-02

**UV-B**	response to abiotic or biotic stimulus	3.1e-12	transferase activity	1.3e-02
	
	response to stress	2.3e-11	kinase activity	1.4e-02
	
	other biological processes	1.6e-08		

**Wounding**	response to abiotic or biotic stimulus	1.4e-17	other enzyme activity	6.3e-06
	
	response to stress	1.0e-15	transferase activity	2.1e-03
	
	other biological processes	1.1e-10	transcription factor activity	2.1e-03
	
	other metabolic processes	4.7e-02	hydrolase activity	1.2e-02
	
			transporter activity	4.1e-02

**Drought**	response to abiotic or biotic stimulus	2.8e-19	structural molecule activity	7.9e-18
	
	response to stress	6.0e-19	transferase activity	6.7e-05
	
	other biological processes	6.4e-10	other enzyme activity	3.8e-04
	
	other metabolic processes	1.8e-02		

**Heat**	other biological processes	7.0e-12	transferase activity	9.3e-04
	
	response to abiotic or biotic stimulus	7.7e-10	other enzyme activity	2.1e-03
	
	response to stress	3.4e-08	hydrolase activity	3.1e-02
	
	electron transport or energy pathways	9.5e-03		

**Oxidative**	response to abiotic or biotic stimulus	7.3e-17	transcription factor activity	2.2e-03
	
	other biological processes	6.5e-14	other enzyme activity	5.7e-03
	
	response to stress	7.7e-10	kinase activity	1.4e-02
	
			transferase activity	1.6e-02

GO-Slim process and function annotation terms enriched in genes with maximal PE under control and treatment conditions. FDR (False Discovery Rate) *p*-values obtained from Fisher Exact tests (see Methods) correspond to the Benjamini-Hochberg-corrected raw *p*-values [47]. Only FDR values smaller than 0.05 are listed.

**Figure 4:**
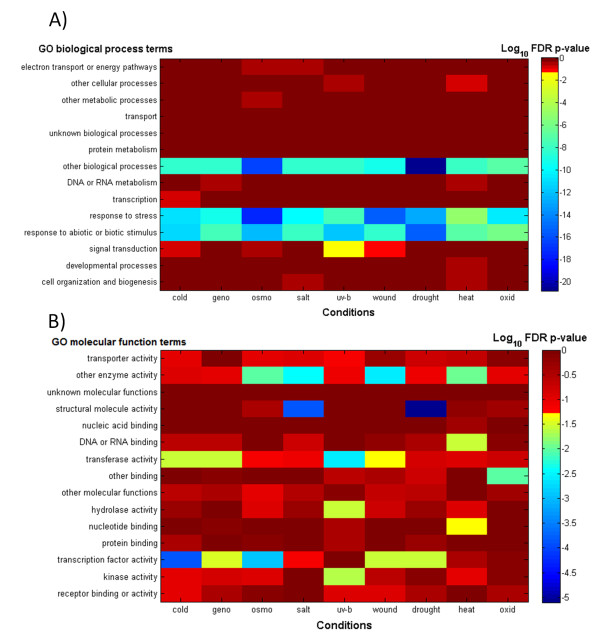
**GO-process (A) -function (B) term enrichment analysis for genes associated with the highest possible *PE *value (*PE *= 2.32) compared to all other genes across all 9 abiotic stress conditions applying Fisher's Exact test**. Temporal expression profiles (*EP *- logarithmic scale) for all genes, *g*, were normalized to control conditions according to ***EP***_N,g _= **EP**_S,g_-**EP**_Ctrl,g _(vector notation), where *S *is a particular stress and *Ctrl, N *denotes control and control-normalized conditions, respectively.

When investigating PE values for temporal expression profiles expressed relative to control values (see legend, Figure 4), we noted that with regard to Biological Process, "response to stress", "response to abiotic or biotic stimulus" and "other biological process" were consistently enriched among genes associated with the highest possible PE value (PE = 2.32) compared to all other genes with lower PE (Figure 4A, blue horizontal stripes), However, with regard to Molecular Function - an ontology that aims more at the precise molecular functionality rather than overall process, no individual function or activity is universally detected as being associated with high PE values upon stress exposures (Figure 4B). Thus, while the overall strategic response is similar across all conditions (stress response), the concrete realization as to which functions are modified by increasing gene expression complexity is condition specific.

### PE and expression level

Evidently, genes with very low or even no appreciable expression levels across all conditions and at all time points will have low (or zero) PE values as there is no significant change either. Among those genes, genes with currently no annotated function or process involvement as judged by their GO annotation are clearly overrepresented (p < < 0.05). It is possible that those genes may be predicted incorrectly in the genome or may even not be true genes at all as frequently gene structure annotations have been generated by *in silico *gene predictions. Thus, including genes with very low expression level bears the risk of associating low PE values simply with genes of questionable gene structure. Therefore, in all subsequent analyses that rely on genes with correctly assigned gene structure and detectable gene expression, we discarded the 10,000 genes expressed at low levels under control conditions (see Methods) and focused on the remaining 11,797 genes with appreciable expression levels only.

### Increased PE correlates with an increased upstream intergenic space and increased number of cis-elements

High PE-values resulting from an increased number of order patterns indicate that a gene's expression has truly changed significantly (according to *d_e_*) over the course of the experiment. And not only has the expression level changed, but it has changed in different directions (successions of up, down, and unchanged regulation) as purely monotonic increases/decreases, despite being above the threshold *d_e_*, will not contribute towards increased PE values. Thus, genes with high PE have undergone differential regulation with opposing outcomes in response to the external stimulus. Naturally, this behavior provokes the question as to how this complex regulation is induced and regulated.

**Figure 5:**
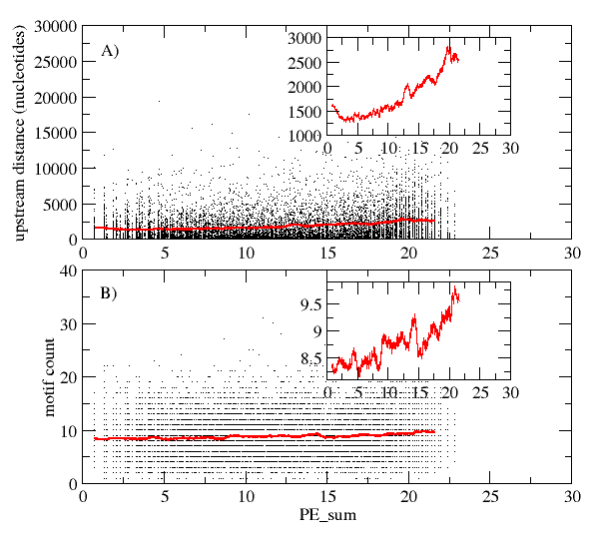
**Relationship between PE values summed up over all experimental conditions (PE_sum) and A) the distance to the next upstream gene (in number of nucleotides), and B) the number of cis-regulatory elements in the 500 nucleotides upstream of the transcription start site**. Raw data (individual gene data) are indicated by dots. The red line corresponds to a 500-point running average of the PE_sum-sorted data. Respective insets show zoomed in views of the running average.

High entropy genes may be under a more complex cis-regulatory control, and thus should harbor more cis-regulatory motifs in their upstream promoter regions. They may also require larger upstream space to accommodate a more complex regulatory apparatus, which would be measurable as the distance to the next upstream gene. Indeed, high PE genes were observed to be associated with significantly longer upstream regions compared to low PE genes. When sorting all genes in ascending order of PE summed up over all conditions (PE_sum), the average upstream-gene-distance for the top 2,000 PE genes was 2,597 +/- 2,481 (s.d., median = 1848) compared to only 1,438 +/- 1,703 (median = 803.5), *p_MWU _*= 4.4e-86 for the bottom 2,000 PE genes. This held also true when considering only control conditions (2,000 high-PE genes: 2,394 +- 2,412 (median = 1645.5), 2,000 low-PE genes: 1,463 +/- 1,643 (median = 888.5), *p_MWU _*= 1.1e-51). As shown in Figure 5A, while the scatter based on per-gene data is appreciable, the average upstream distance shows a consistent upward trend with increasing values of PE_sum (inset of Figure 5A).

We then examined the number of cis-elements annotated within the 500 nucleotides upstream of a gene's transcription start site. High-PE genes were found to be associated with a significantly increased number of cis-elements, albeit the absolute difference was small (around 10%). The 2,000 highest PE genes possess, on average, 9.4 +/- 3.9 (median = 9) cis-elements compared to 8.4 +/- 3.6 (median = 8) found in the 2,000 lowest-PE genes, *p_MWU _*= 6.2e-16. Figure 5B shows the relationship of motif counts and PE_sum as a scatter plot. As for upstream distances (Figure 5A), the per-gene data vary substantially, but a consistent upward trend of motif counts with increasing PE_sum values is clearly discernable (inset of Figure 5B). The trend of increased cis-element motif counts with increased PE was observed similarly for unique motif counts; i.e., repeated occurrences of the same motif were not counted towards the total number of motifs.

### Cis-regulatory elements associated with high and low PE genes

We determined if and which motifs are overrepresented in high- or low-PE genes. Significantly increased motif counts for particular motifs were found for both high and low PE genes (Table 3). Well known stress-response cis-regulatory motifs such as the ABRE-like and ABF binding site motifs [31] were found overrepresented in upstream regions of high-PE genes, while in upstream regions of low-PE genes, the TELO-box motif known to control genes involved in house-keeping functions [32] was identified as the most significantly overrepresented motif.

**Table 3:** Cis-regulatory motifs associated with high and low PE genes.

High PE		Low PE	
**FDR p-value**	**Motif**	**FDR p-value**	**Motif**

1.29e-33	ABRE-like binding site motif	1.65e-17	TELO-box promoter motif

3.04e-28	ACGTABREMOTIFA2OSEM	1.51e-04	GAREAT

2.63e-23	CACGTG MOTIF	1.69e-04	CARGCW8GAT

5.58e-16	ABFs binding site motif	6.21e-04	MYB4 binding site motif

6.79e-14	GBOXLERBCS	1.35e-03	MYB1AT

6.31e-10	EveningElement promoter motif	3.44e-03	T-box promoter motif

1.09e-07	TGA1 binding site motif	5.60e-03	L1-box promoter motif

2.80e-07	GBF1/2/3 BS in ADH1	1.06e-02	Gap-box Motif

1.14e-06	ABREATRD22	1.25e-02	E2F binding site motif

1.07e-05	DRE core motif	1.25e-02	E2FAT

8.21e-05	Ibox promoter motif	1.25e-02	MYB2AT

2.44e-04	UPRE2AT	3.88e-02	CCA1 motif1 BS in CAB1

2.69e-04	UPRMOTIFIIAT	4.25e-02	MYB1LEPR

8.96e-04	DREB1A/CBF3		

Cis-regulatory motifs found overrepresented with Benjamini-Hochberg corrected *p*-values [47]*p_FDR _*< 0.05 in the 500 nucleotides upstream of the 2,000 high and low PE genes, respectively.

### Genes with high connectivity degree in correlation networks have increased PE

**Figure 6:**
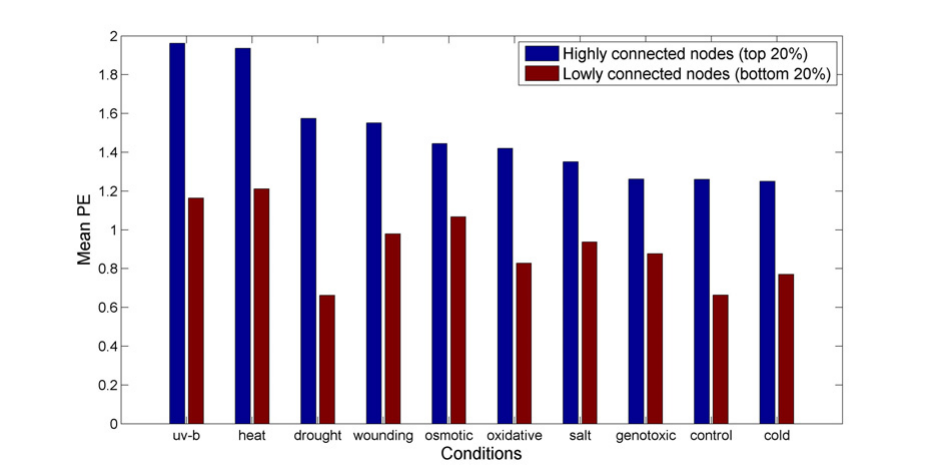
**Average PE associated with high (top 20% of all gene transcripts) and low (bottom 20%) degree correlation network connectivity degree (see Methods) across all experimental conditions**. Only gene transcripts with expression level rank greater than 10,000 in the control samples have been considered in the analysis to account for possible expression level biases. (Similar results were obtained, when all genes were included with high degree genes on average being associated with increased PE values, Additional File 1, Supplementary Figure S2).

The PE measure characterizes the temporal expression profiles of single genes. Next, we explored the PE of genes in relation to their respective network properties by way of comparing high to low degree genes, where degree measures the number of connections in correlation networks (see Methods), thus embedding single PE values in the context of other genes. Across all conditions, we found that highly connected genes exhibit increased PE values relative to genes with low degree (Figure 6). Thus, while simple expression profiles dominate - about half of all genes show the lowest PE value under control conditions (Figure 2), with regard to similarity of temporal profiles as judged by correlation, more complex patterns seem to be adopted synchronously by larger numbers of genes.

### Conserved genes have lower PE compared to new, Arabidopsis-specific genes

For the set of 11,797 high expression level Arabidopsis gene probes (see Methods), we identified those genes that have been conserved over long evolutionary times - and thus can be assumed to be evolutionarily old - by comparing all Arabidopsis gene-encoded protein sequences to all annotated protein sequences in *Physcomitrella patens*, a moss, and *Chlamydomonas reinhardtii*, a unicellular algae. The 5,012 Arabidopsis genes also contained in Physcomitrella were found to have significantly lower mean PE when summed up over all conditions (< PE_sum _> = 11.1 +/- 6.0 (s.d.), median = 10.7) compared to 6,785 genes only present in Arabidopsis, and thus presumably evolutionarily young genes (< PE_sum _> = 11.9 +/- 6.4, median = 12.1), *p_MWU _*= 1.3E-13. Similarly, the 1,647 gene found both in Arabidopsis and Chlamydomonas are also associated with decreased PE <PE_sum _> = 11.0 +/- 5.8, median = 10.7 versus <PE_sum _> = 11.7 +/- 6.3, median = 11.6 for 10,150 Arabidopsis-specific genes, *p_MWU _*= 4.6E-05.

## Discussion

Typically, the notion of complexity is used in the context of entire systems, for example protein-protein interaction networks [33]. We introduced the concept of Permutation Entropy (PE) to capture the complexity of a singular temporal expression profile. Here, complexity may be understood in the Kolmogorov Complexity [34] sense, namely as the quality of a profile of being simple (unchanged or monotonic) as opposed to more complex (succession of up and down) to that of highest complexity (a situation in which a profile can not be reduced to a simple generating principle such as a linear of periodic function; i.e. that of highest entropy). Specifically, we applied the PE metric to the analysis of temporal gene expression profile data. It relies on decomposing sequential expression patterns into elementary motifs (ordinal motifs) and associates high complexity with high and low complexity with low numbers of different motifs. Investigations of time series data have focused primarily on discerning differential gene expression; i.e., the discovery of genes whose expression is significantly altered in response to a perturbation by applying appropriate thresholds. As implemented here, PE also identifies patterns of differential expression - or absence thereof (no-change pattern), but in addition, examines whether the response is 'simple' (e.g. monotonic) or whether a succession of different motifs indicates more complex transcriptional changes. Thus, PE allows to easily identify those transcripts that exhibit intricate expression profiles as candidates for complex regulatory actions.

The issue of complexity of individual series of level data has been addressed before, most notably by Ahnert and co-workers, who also used a pattern-based approach associated with rank permutations [20]. In the PE formulation of the problem applied here, a single mapping function (Eq. 1) to map series of rank numbers to single values is used and thus to associate a single quantity with each temporal profile rather than several possible mapping functions listed in [20]. Furthermore, by introducing the no-change pattern that is supposed to capture level differences below the noise level, we specifically aimed to associate those profiles to high complexity that are characterized by an occurrence of many different up/down/unchanged patterns, whereas in [20], no such cutoff was introduced, such that seemingly erratic up/down patterns would be identified as random, and thus of lesser biological significance. By applying a sensible noise cutoff, we intended to identify those profiles as highly complex that are likely to have resulted from many and significant changes of the transcriptional program acting on those transcript. By contrast, the approach used in [20] primarily aims to identify profiles that are most non-random (as opposed to most complex as defined here using PE-complexity) and thus those profiles that are associated with a particular biological process. For example, in our PE approach cyclical patterns obtained for cell cycle series would be ranked as medium PE complexity as repetitive patterns have less than maximal PE, but as most highly non-random by the approach introduced in [20]. In that regard, our PE formalism detects complex profiles as those with many significant changes and - to some extend - independent of the actual experiment.

As a metric, PE copes with the three problems typically encountered by other complexity measures (see Introduction). It is applicable to short, non-stationary, and non-equidistant time series data. Evidently, longer time series will, however, yield statistically more robust results on a per gene basis.

As all studies on dynamic processes, the sampling rate at which data points are taken critically influences the results and their interpretation. Ideally, the sampling rate matches the characteristic time scale of the process under investigation. The data used in this study were spaced in the minutes and hours time interval range, a scale that was found appropriate for transcriptional responses. It is clear that, had the sampling frequency been much higher or lower, the absolute PE values may not be directly comparable across different sampling frequencies. For example, at much higher rates, the unchanged pattern will be observed much more frequently. However, in relative terms, i.e. a comparison of PEs associated for a number of genes at a given sampling scheme, the ordering of genes with regard to PE can be expected to hold. Furthermore, the data used here were spaced evenly on a logarithmic time scale. By applying interpolation using cubical splines, we simulated even linear time spacing and found very similar results with regard to the overall distribution of PE values (Additional File 1, Supplementary Figure S3) and qualitative results with regard to GO functional annotations (Additional File 1, Supplementary Figure S4). Thus, the PE metric proved robust with regard to sampling scheme details.

### Permutation Entropy and noise

High complexity measured by high PE indicates low predictability of future expression values based on past values of a gene expression time series. Noisy or very erratic gene expression profiles would also qualify as highly entropic and, thus, not predictable. By imposing a threshold between consecutive time points based on the observed technical and biological variation, we largely eliminated purely noise-induced expression changes and identified significant change patterns. Thus, high PE values can be assumed to be the result of significantly altered transcription programs and not by technical or biological noise. However, the appreciation of a biological role of noise in generating physiological responses is only now emerging. Recently, it was shown that noise may produce bistable positive transcriptional feedback loops [35] and that furthermore noisy genes have conserved coding sequences and exhibit characteristic protein-protein interaction network properties [36]. The concept of PE may thus be a suitable metric to be used in such studies as well.

### Mechanisms of gene expression complexity

As we implemented the PE concept to specifically identify patterns of significant change, the question arises as to how successions of multiple up/down/unchanged patterns for a single gene in response to a single stimulus are regulated. We found evidence that high PE genes are characterized by an increased length of their 5'-intergenic region as well as an increased density of cis-regulatory motifs. Thus, increased PE may in part be originating from larger and more complex promoter regions. This has been shown similarly in the context of multi-stress response genes signified by differential expression across different external perturbations contained in the AtGenExpress abiotic stress experiment series that was also used here [37]. However, we took a different view by examining the complexity of temporal profiles of individual genes in a single stress experiment. As we find similar trends, cross-experiment complexity and temporal single-experiment complexity may have similar sources based on genomic, architectural properties, and cis-regulatory motifs. The role of other conceivable processes and factors involved in causing highly complex temporal expression profiles such as miRNA, epigenetic, chromosome structurally mediated regulations [38] remains to be established.

### Complexity and evolution

Including only genes with appreciable expression levels under control conditions, we found that Arabidopsis genes that are conserved over long evolutionary distances, which can be assumed ancient, have lower PE than Arabidopsis specific genes. While the absolute difference was small (around 7%), it was statistically very significant. It appears reasonable to conclude that conserved genes serve functions that are universally needed. Indeed, among the conserved genes (both Arabidopsis vs. Chlamydomonas and vs. Physcomitrella, genes involved metabolic and protein metabolic processes, cell organization and biogenesis, and electron transport or energy pathways were overrepresented as judged by a GO-term enrichment analysis. Genes specific to Arabidopsis emerged later in evolution to potentially cope with more complex environments, and thus needed more complex gene expression regulation. In this study, complexity of the environment has essentially been mimicked by the different stresses applied. In the Arabidopsis specific gene set, genes involved in transcriptional regulatory processes were found overrepresented as well as genes with currently unknown function or process involvement. As has been emphasized [26], deciding the question of increasing complexity on evolutionary time scales may to a large degree depend on the definition of complexity. Here, we used PE as an entropy-based measure to capture complexity.

### Permutation Entropy and the abiotic stress response in Arabidopsis

When challenged by environmental perturbations the transcriptional programs are being modified not only to change the expression levels of genes, but the adjustments is accompanied by inducing and repressing interactions leading to increase complexity of temporal expression profiles. Among the different abiotic stress conditions, exposure to heat and UV-B light stress conditions provoked the greatest changes of PE. Thus, both conditions appear to present major assaults resulting in massively change transcriptional programs. At the other end of the spectrum, cold stress - while also representing a temperature cue - resulted in relatively small PE changes. Intuitively, this could be interpreted as a general thermodynamic "freeze-up" generating responses of smaller amplitude. Whether this amplitude reduction is indeed a thermodynamic effect or a result of regulation remains to be established. In this context, it would be interesting to monitor concurrent metabolic changes as the temperature sensitive reaction rates would also reveal thermodynamic effects.

Genes involved in stress response processes and other abiotic stimuli were found to exhibit greatest PE. Surprisingly, however, even under reference conditions, genes annotated as being involved in stress response have increased PE relative to other gene functions (Table 2). Of course, it is difficult to decide what actually constitutes a stress condition. Also the reference, control condition is one particular set of environmental parameters the plant has to cope with. Growth processes and circadian as well as diurnal rhythms persist requiring dynamic changes of the underlying transcriptional program of those genes. Alternatively, it could be speculated whether those genes are naturally fluctuating more, such that they are active and not in a dormant state, ready to produce the necessary response when challenged.

We found that the overall strategy of stress response may be the same across different abiotic stresses - to cope with the stress condition (Figure 4A), the concrete tactics as to how to accomplish the stress response and which particular functional program to change, may very much be stress specific (Figure 4B).

Our analysis furthermore highlights the importance of time-resolved studies of stress response dynamics. Approached naively, a stress condition should induce or repress a gene's expression and lead to a one-time or steady response pattern. Correspondingly, previous analyses on the abiotic stress response in *Arabidopsis thaliana *using the same dataset as analyzed here were based on detecting differential expression at a selected, single time point [37]. Here, we show that oftentimes stress response is signified by a complex pattern of up- and downregulation instead. Thus, when aiming to investigate stress response, single or few measurement time points may fall short of unraveling the true stress response dynamics. In addition, simple fold change considerations may be augmented by metrics that capture the complexity of response as well, as the one introduced here, the Permutation Entropy.

Although the focus here has been on the characterization of single profiles, the reduction of continuous level data to patterns or symbols has also been explored in the context of modeling dynamical systems including interactions between many interacting network constituents [39]. The applicability of the PE metric to capture interactions may thus offer a fruitful avenue for further research.

## Conclusions

Permutation Entropy provides a simple, yet powerful metric to capture complexity in patterns found in temporal profile data of single entities such as gene transcripts or individual metabolites. While longer time series data are preferable, even for relatively short time series with only few data points, meaningful results can be obtained. Evidently, the concept of PE lends itself to the analysis of any time series data. As generating time series is becoming increasingly common, PE may emerge as a standard quantitative approach for their analyses.

## Methods

### Expression data, Probe Set

*Arabidopsis thaliana *gene expression time series data based on the Affymetrix ATH1 microarray available as part of the AtGenExpress data resource [30] were obtained from The Arabidopsis Information Resource - TAIR [40,41]. The dataset comprised nine abiotic stress (including heat, cold, genotoxic, UV-B-light, osmotic, salt, wounding, drought, and osmotic stress conditions) and one common control condition. All raw hybridization data (CEL-files) for each condition were processed and RMA-normalized [42] using Matlab 7.6.0.324 (R2008a) Bioinformatics Toolbox (version 3.1). Repeat hybridizations were averaged and all data log_2_-transformed. In total, expression data for 22,746 Arabidopsis gene probes measured at 7 time points common to all conditions and corresponding to 0 h, 0.5 h, 1 h, 3 h, 6 h, 12 h, and 24 h after stress induction were used for the analysis. Only ATH1 probes with unique gene-probe mappings and those mapping to nuclear encoded genes were used, leaving 21,797 gene probes for analysis.

### Modified Permutation Entropy (PE) algorithm tailored to gene expression time series data

Permutation Entropy (PE) as introduced in [23] was applied to the Arabidopsis gene expression time series data investigated here. In short, PE corresponds to the entropy contained in a gene's, *g*, temporal expression profile based on the probabilities of possible orderings, or order patterns of time points sorted by expression value, of *n *subsequent data points, with *n *set to three, according to Eq. 1:

(1) $$PE_{g}=-\sum_{i=1}^{N}p_{i}\log(p_{i}),$$

where *p_i _*is the estimated probability of occurrence of order pattern *i *in the *t *= 7 time series data points measured for gene *g *under a particular experimental condition and computed as the relative frequency of pattern *i *in the total of 5 consecutive three-point order patterns (*t*-*n*+1 patterns are possible in *t *time points). The summation is performed over all motifs actually observed in a given gene's time series. As there are *N *= 13 different three-point order pattern (see below), 7 discrete PE values are possible (corresponding to these discrete possible realizations 5 (all patterns identical), 4 (the same)-1 (different), and likewise: 3-2, 3-1-1, 2-2-1, 2-1-1-1, 1-1-1-1-1, resulting from 5 consecutive order patterns. Thus, the lowest possible PE value for a gene is zero - only one order pattern is realized - while the maximal possible PE value is PE_max _= -5*1/5*log_2_(1/5) = 2.32 corresponding to five different patterns with equal probability.

Caused by technical noise and biological variability, small expression differences may not reflect an actual and significant change, but rather essentially unaltered expression levels. Therefore, deviating from the original PE computation procedure, we introduced the "no-change" pattern to avoid overly associating complexity (increased PE) with pure noise. A gene's expression level in two consecutive time points was regarded as unchanged if the respective difference was below a specified threshold, *d_e_*, set to *d_e _*= 0.35 corresponding to the determined mean absolute difference (*d_m_*) plus one standard deviation between repeat measurements across all datasets with *d_m _*= 0.156 ± 0.19 (log_2 _values). By introducing the no-change pattern, *N *= 13 different order motifs for three consecutive time points (n = 3) are possible. Similar results were obtained for different values of *d_e_*.

**Figure 7:**
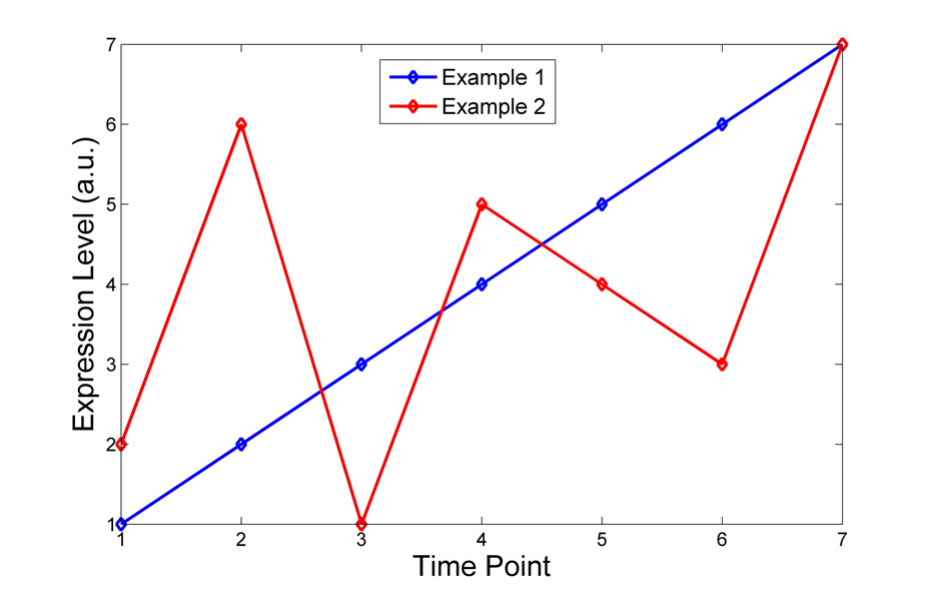
**Illustration of the concept of Permutation Entropy (*PE*)**. The two artificial, exemplary gene expression time-series profiles in arbitrary units (a.u.) have the same length, but the resulting PE is different. For Example 1, only one order pattern is observed, namely (1,2,3); i.e., all values are strictly in ascending order. According to Eq. 1, its associated PE computes as PE_1 _= -5/5*log_2_(5/5) = 0. In Example 2, five different order patterns are observed (sliding window of three consecutive expression values): (2,3,1), (3,1,2), (1,3,2), (3,2,1), and (2,1,3). The position in each triplet corresponds to the temporal order, the associated value to the rank of corresponding expression value within the triplet, and correspondingly PE_2 _= -5*(1/5)*log_2_(1/5) = 2.32. Thus, Example profile 2 has a greater PE complexity compared to profile 1. The selected cases illustrate the extreme values of 0 and maximal PE of 2.32. There exists a certain degeneracy, which means that the PE for two genes may be the same even though their ordinal pattern sequences are different. For example, a gene may exhibit a temporal motif sequence of 1+3+6+7+9, while another gene followed a 2+5+10+12+9 pattern sequence, their entropy values are the same (*PE *= 2.32; i.e., the maximally possible value corresponding to 5 different patterns). Consequently, only seven discrete PE values are possible.

The computation of PE is illustrated schematically for a time series consisting of 7 time points in Figure 7 and all possible order patterns are shown in Figure 1A.

### Gene Ontology annotation

Gene Ontology (GO) slim term based gene annotation information was obtained from TAIR [40,41]. All Arabidopsis genes were grouped into 14 GO process- and 15 molecular function categories according to the defined GO-slim terms.

### Identification of genes with appreciable expression level

Low PE-values would also be obtained for genes not being expressed at all (or expressed at very low levels) across all time points and across the various conditions as there is no detectable change either. To eliminate this trivial result and to focus on genes whose expression level is indeed detectable, we sorted all gene probes based on their expression levels in the control sample and discarded the 10,000 genes with lowest expression rank from the analyses pertaining to motif counts, intergenic distances, and conservation. For the remaining 11,797 gene probes, no dependency of their cumulative PE (PE values summed up over all conditions) on expression rank was observed, while it increased steadily with increasing rank below the 10,000 rank cutoff (Additional File 1, Supplementary Figure S1). Thus, possible effects of absolute expression level on PE (as the trivial no expression = zero PE relationship) have been reduced by eliminating the 10,000 genes expressed at low levels under control conditions.

### Cis-regulatory motif information, Distance to next up-stream gene

Cis-regulatory motif annotation information associated with all Arabidopsis genes was obtained from the Athena database [43] and processed as described in [37]. Cis-regulatory motifs were considered up to 500 nucleotides upstream of a gene's annotated transcription start site. Only genes with intergenic upstream regions larger than 500 nucleotides were considered in the associated analyses. All motifs mapping to the considered regions were considered; i.e., multiply mapping motifs were not rendered unique. Motifs were allowed to overlap. Intergenic distances were obtained from TAIR [40,41].

### Identification of conserved plant genes

Well conserved ('ancient') plant genes across long evolutionary times were determined by identifying all genes shared (conserved) between *Arabidopsis thaliana and Physcomitrella patens*, a moss, and *Chlamydomonas reinhardtii*, a single-cell algae. All 30,690 Arabidopsis protein sequences obtained from TAIR [40,41] were aligned to all 35,938 protein sequences from Physcomitrella [44] and all 16,709 protein sequences annotated in Chlamydomonas [45] (both protein sequence sets obtained from the Joint Genome Institute, JGI http://www.jgi.doe.gov/) using *blastp *[46]. Pairwise alignments with greater or equal to 50% sequence identity over an alignment length of at least 100 amino acids were considered to be associated with conserved proteins, and in turn, genes. In total, 6,678 proteins/genes were identified as conserved between Arabidopsis and Physcomitrella, and 2,074 proteins shared in Chlamydomonas and Arabidopsis according to the applied parameters. Note that all reported results are obtained similarly, when a more stringent 70% sequence identity threshold was applied. Furthermore, in the analyses reported under Results, only those genes with high expression levels were included (see above).

### GO-term enrichment analysis

GO-term enrichment analyses were performed using the Fisher's exact test. False Discovery Rate (FDR) multiple testing correction was applied to the obtained p-values according to Benjamini-Hochberg [47].

### Correlation network connectivity degree

Across all experimental conditions, all temporal gene expression profiles were correlated using the Pearson correlation coefficient, *r*, in an all-against-all fashion. Two gene transcripts were considered connected if their respective pairwise absolute correlation coefficient, *r*, was greater than 0.98. The degree of a gene transcript refers to its summed up number of connections.

## Authors' contributions

XS and DW designed all tests and analyses and performed all computations. YZ and JK introduced the concept of PE and contributed to the interpretation of results. VN initiated the research into the complexity of temporal gene expression profiles. XS, YZ, JK, and DW wrote the manuscript. All authors read and approved the final version of the manuscript.

## Supplementary Material

Additional file 1 **Supplementary Figures**. Contents: Supplementary Figures S1, S2, S3, S4 Click here for file
