# Supplementary material for: The complexity of gene expression dynamics revealed by permutation entropy
Source: BMC Bioinformatics. 2010 Dec 22;11:607. doi: 10.1186/1471-2105-11-607 (PMC3098107; doi:10.1186/1471-2105-11-607)
Supplement: Additional file 1 — Supplementary Figures. Contents: Supplementary Figures S1, S2, S3, S4 [file 1471-2105-11-607-S1.DOC]

**Additional Material, Sun et al.**


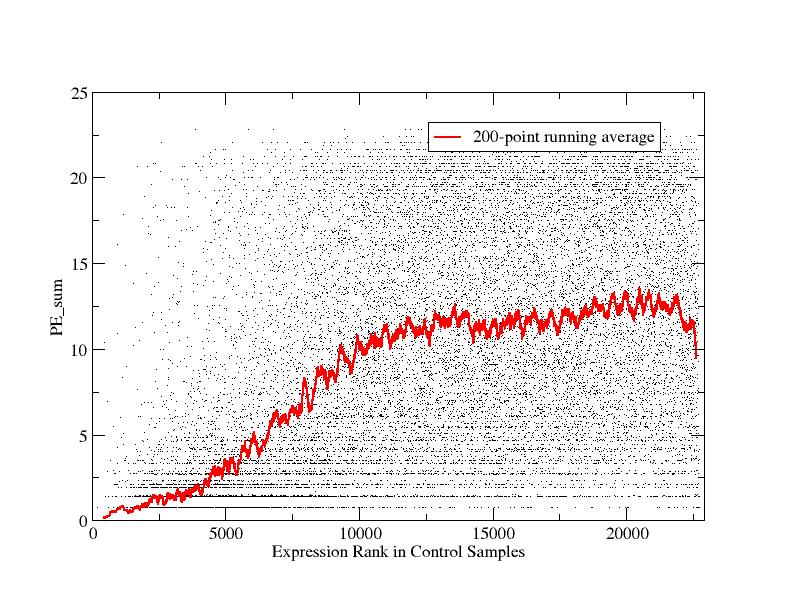
**Supplementary Figure S1** Permutation Entropy values summed up over all experimental conditions (PE_sum) for all gene probes on the ATH1 Affymetrix microarray as a function of their expression level under control conditions. Expression levels of all gene probes were sorted in ascending order and are represented in the graph as associated ranks.

**
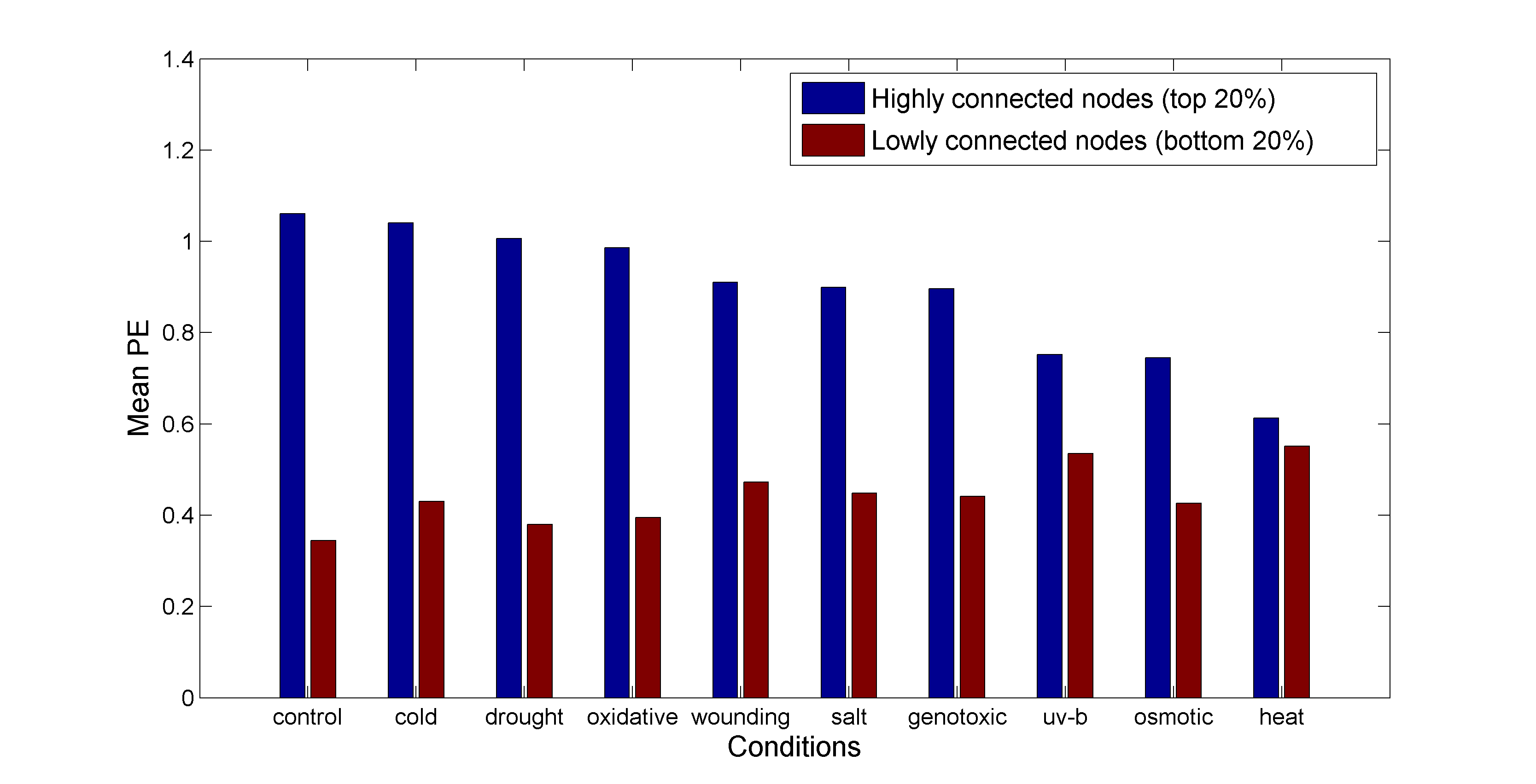
**

**Supplementary Figure S2.** Average PE associated with high (top 20% of all gene transcripts) and low (bottom 20%) degree correlation network connectivity degree (see Methods) across all experimental conditions and for all gene transcripts.


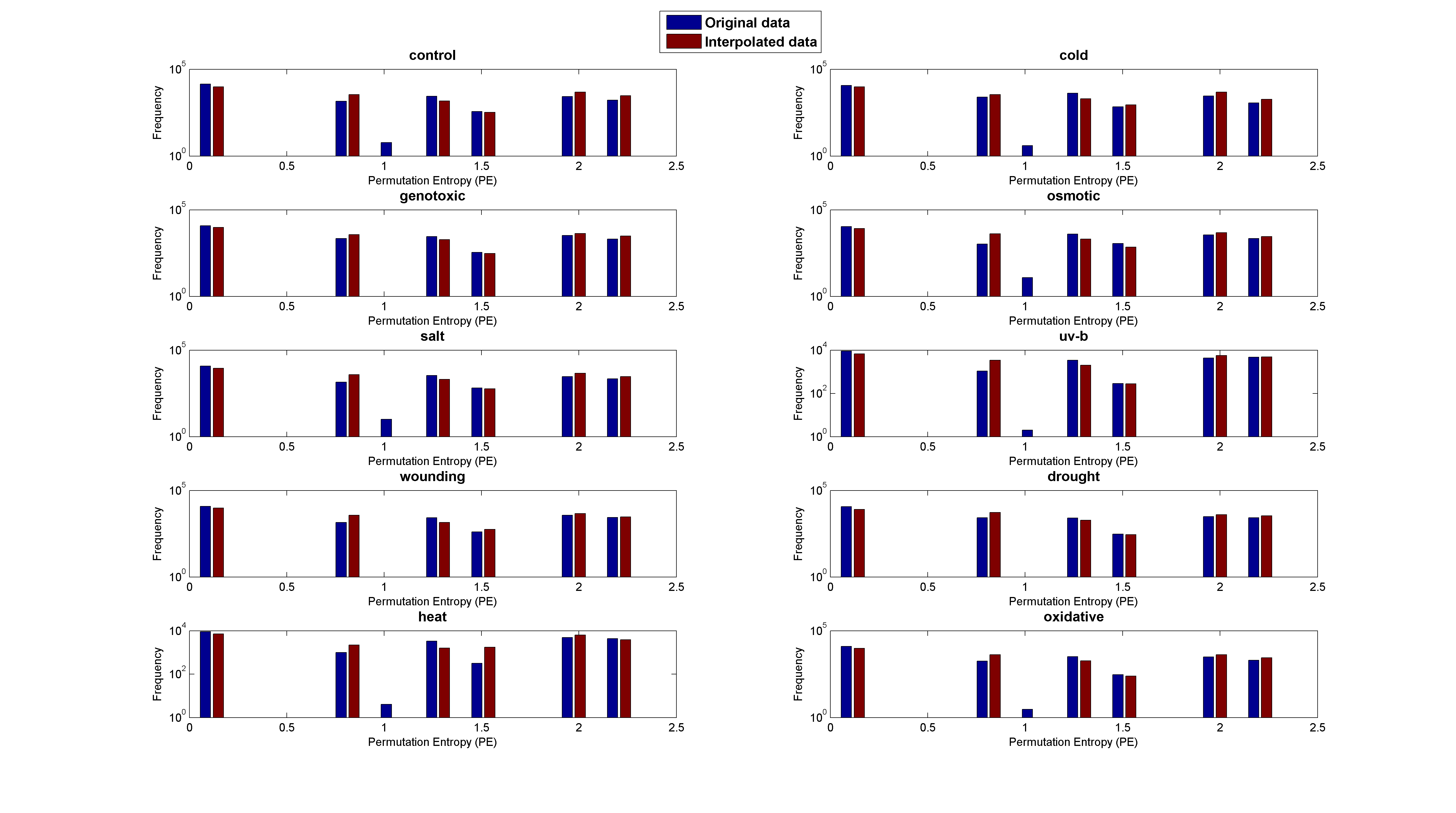


**Supplementary Figure S3.** Comparison of PE frequency distributions across all conditions between the original sampling scheme (close to logarithmic temporal spacing: ([0, 0.5, 1, 3, 6, 12, 24] hours) and linear spacing obtained from cubic-spline-based interpolations: [0, 4, 8, 12, 16, 20, 24] hours.

A)


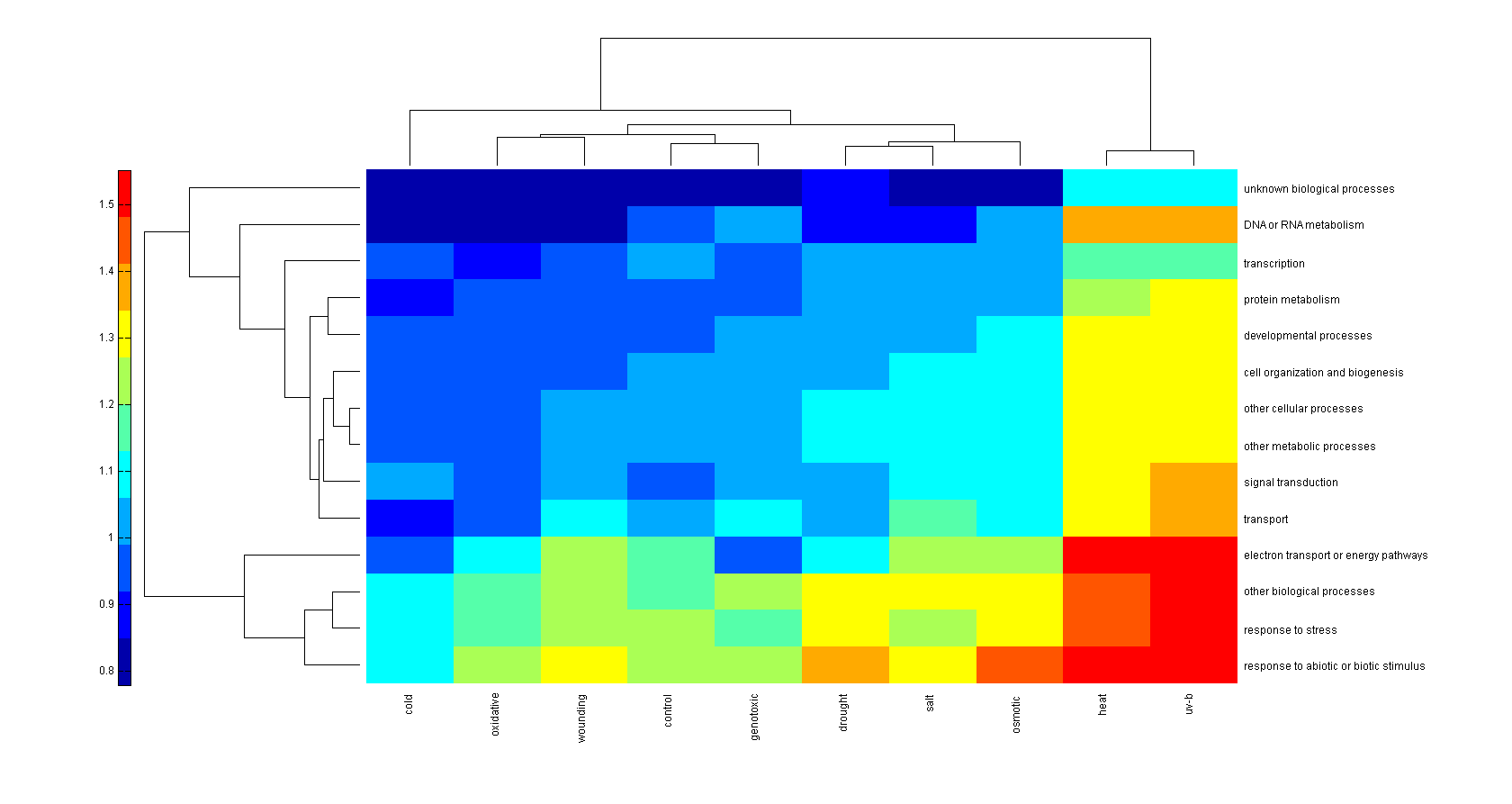


B)


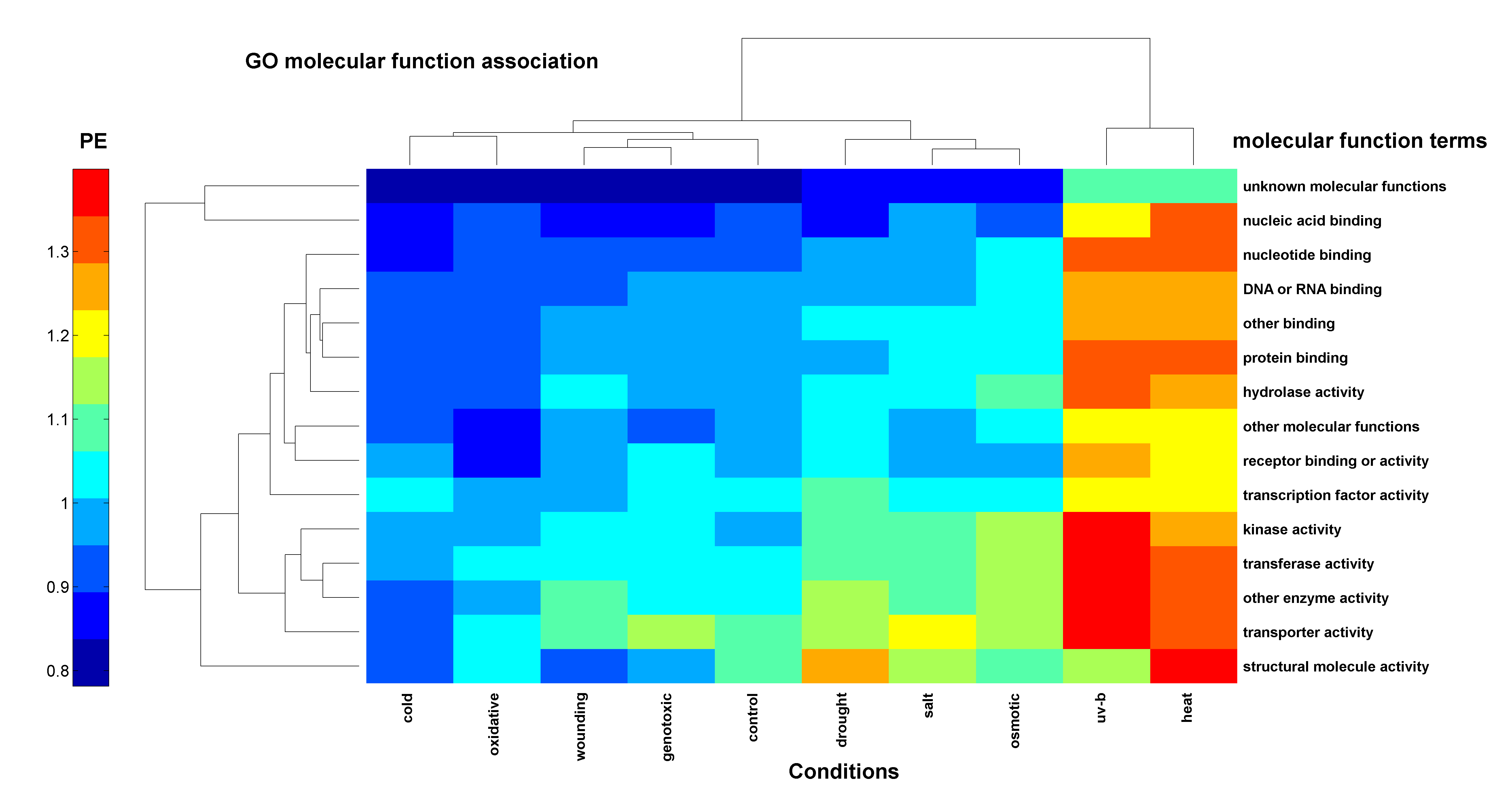


**Supplementary Figure S4.** Biclustering (average linkage) of the mean *PE* computed for gene sets grouped by their GO-Slim biological process (A) or function (B) annotation across all nine abiotic stress conditions and the control condition and for the interpolated linear temporal spacing obtained from cubic-spline-based interpolations: [0, 4, 8, 12, 16, 20, 24] hours. Results are similar to the ones obtained for the original temporal spacing (Figure 3, main manuscript).
